# “It was up to me to be curious”: perceptions and experiences of students with intellectual disability on genetics and health education

**DOI:** 10.1038/s41431-026-02041-w

**Published:** 2026-02-23

**Authors:** Jennifer Hansen, Iva Strnadová, Joanne Danker, Karen-Maia Jackaman, Julie Loblinzk Refalo OAM, Skie Sarfaraz, Jackie Leach Scully, Jackie Boyle, Bronwyn Terrill, Elizabeth Emma Palmer

**Affiliations:** 1 School of Education, UNSW Sydney, Sydney, NSW Australia; 2 Disability Innovation Institute, UNSW Sydney, Sydney, NSW Australia; 3 Self Advocacy Sydney Inc, Sydney, NSW Australia; 4 Discipline of Paediatrics and Child Health, School of Clinical Medicine, Faculty of Medicine and Health, UNSW Sydney, Sydney, NSW Australia; 5 Genetics of Learning Disability Service, Hunter New England Health, Waratah, NSW Australia; 6 Clinical Translation and Engagement Platform, Garvan Institute of Medical Research, Sydney, NSW Australia; 7 School of Clinical Medicine, St Vincent’s Clinical Campus, Faculty of Medicine and Health, UNSW Sydney, Darlinghurst, NSW Australia; 8 Australian Genomics Health Alliance, Melbourne, VIC Australia; 9 Sydney Children’s Hospitals Network, Randwick, NSW Australia

**Keywords:** Public health, Patient education

## Abstract

People with intellectual disability want to learn more about their health and genetics. They want to be empowered with the knowledge and skills to make informed health and genetic healthcare choices. Little is known about what high school students with intellectual disability learn about health, genetics or genetic healthcare. To address this gap, we conducted an inclusive qualitative research study in Australia. Fourteen Australian current and recently graduated students with intellectual disability participated in semi-structured interviews. Inductive content analysis revealed four key themes: (i) Science, health and genetics education, (ii) Health rights, (iii) Education rights, (iv) Recommendations for improving genetic and health literacy. Students with intellectual disability reported they were not taught about genetics and health, making healthcare choices, and/or making life decisions at school. They felt these disadvantages and were disempowered in becoming informed healthcare consumers. They recommended that teachers should be supported with resources to deliver inclusive, person-centred and respectful lessons that inform decision-making about genetics, health and healthcare choices. Future research should focus on how best to upskill teachers to support students with intellectual disability for their future health choices in a respectful, supportive, student-centred and strengths-based way. This will help prepare young people with intellectual disability to navigate the healthcare system and be empowered partners in their own healthcare. The growth of such skills has been suggested as critical in inequities in healthcare access for people with intellectual disability, as well as improving healthcare experiences and outcomes.

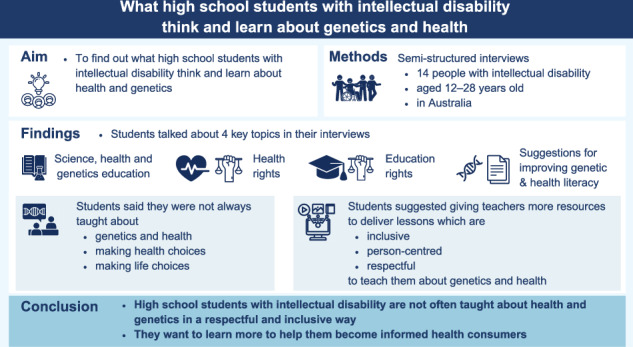

## Introduction

Health literacy is considered a critical determinant of health [1]. People with intellectual disability experience premature mortality, more avoidable deaths and have poor healthcare experiences [2, 3]. Australia’s National Roadmap for Improving the Health of People with Intellectual Disability acknowledges these inequities and emphasises the need for collaborative action to address health disparities [4]. Recent studies identified the ability to access, understand, appraise and use health information and apply it to make informed decisions about health and healthcare as a key barrier to high-quality healthcare for people with intellectual disability [5].

Clinical genetics is revolutionising our understanding of the causes of numerous conditions, including cancers, renal conditions, and many types of intellectual disability [6]. Genetic diagnosis can lead to better physical and mental health outcomes by providing more precise information to guide treatment and empowering patients about future health and reproduction [7]. However, people with intellectual disability may miss out on these opportunities due to inequitable and non-inclusive genetic healthcare [8, 9]. Recent research shows that people with intellectual disability both want and have capacity to learn about their own genetics [10, 11].

Genetic health literacy is defined as “sufficient knowledge and understanding of genetic principles for individuals to make decisions that sustain personal well-being and effective participation in social decisions on genetic issues” [12]. This can incorporate, for example, understanding potential advantages and disadvantages of genetic tests and gene-based therapies, and knowledge to make fully autonomous or shared genetic healthcare decisions. Genetic literacy can also include recognising and managing uncertainty because genetic science is emerging and the meaning of genetic information can be complex and change over time [13]. As genetics is integrated into healthcare, equitable access to support and education to develop genetic health literacy is needed for all members of society, with reasonable adjustments according to each individual’s learning and communication abilities [14]. Becoming an empowered genetic health ‘consumer’ requires education and support in two main areas: ‘functional’ or foundational genetic health literacy, including knowledge of genes, genetic inheritance patterns, chance of a genetic conditions; and growth of skills and confidence to identify and advocate for one’s own healthcare goals and psychosocial needs in relation to genetic healthcare. This requires knowledge of one’s healthcare rights and responsibilities and skills in self-advocacy and self-management [15]. This area has been described as encompassing ‘interactive’ and ‘critical’ health literacy [16, 17].

In Australian schools, genetics-related content is predominantly delivered in the Science syllabus [18]. The high school science curriculum lays the foundations of genetic knowledge to support the development of genetic health literacy by including: DNA structure and function, how traits are inherited, and the link between genetic variation and conditions such as cancer, heart disease and diabetes [19–21]. The curriculum also contains content on how advances in technology can solve healthcare challenges and how people may use different ways of decision-making when deciding whether to apply new technologies to their own healthcare [21].

There is little research on how to best teach students with intellectual disability about health and healthcare, including self-advocacy and self-management generally, and genetics and genetic healthcare specifically. In a recent qualitative study, we identified gaps in teachers’ knowledge of how to teach students with intellectual disability about genetics and health literacy in an accessible way, and a lack of resources to help teachers apply evidence-based practices in health and genetic education [22].

This study aimed to explore the perceptions and experiences of students with intellectual disability with respect to genetics and health education. To date, no study has focused on education in genetics, health, and health literacy from the point of view of students with intellectual disability. The research question guiding this study was: What are students’ perceptions and experiences of high school education relevant to health and genetics?

## Methods

This was an inclusive qualitative research study. In inclusive research, people with disability are not just participants but also co-creators of knowledge [23]. Inclusive research involves co-production, a collaborative process where all stakeholders are involved in knowledge creation, fostering a shared community of practice [24]. The authors consist of academic researchers from diverse disciplines (special education, disability studies, bioethics, genetics) and three co-researchers with intellectual disability All study materials, including participant information statement and consent forms (PISCF) were available in Standard and Easy Read English. The study was approved by UNSW Sydney’s Human Research and Ethics Committee (HC210342).

### Reflexivity statement

This research was conducted by a neuromixed team that included academic researchers from special education, disability studies, bioethics, and genetics, as well as three co-researchers with intellectual disability who were integral to all stages of the research process. Our diverse positionalities – encompassing both professional expertise and lived experience of intellectual disability – shaped our approach to the research questions, methodology, and data interpretation. The academic researchers brought varied disciplinary perspectives on inclusive education, health literacy, and genetics, while the co-researchers contributed essential insights from their experiences as people with intellectual disability navigating health and genetics education. This collaborative approach enabled us to centre the voices and experiences of people with intellectual disability throughout the knowledge creation process, aligning with inclusive research principles and the ‘Nothing about us without us’ framework that guided this study.

### Participants

Individuals aged 12–28 years with intellectual disability and living in Australia were recruited through social media channels, including X (formerly Twitter), Instagram, Facebook and LinkedIn, professional networks, parental networks, and grassroots organisations (e.g., self-advocacy organisations). A social media flyer included an introduction to genetics and health taught at school, the study aim, inclusion criteria and contact details.

### Interviews

The semi-structured interview protocol was co-developed by a multidisciplinary team including researchers with expertise in special education and disability studies, a clinical geneticist, and co-researchers with intellectual disability. Co-researchers are an integral part of the GeneEQUAL research program and are involved in all meetings and each step of the research process. Interviews were conducted in person or online via Zoom. Consent was provided by participants using an accessible Easy Read PISCF, and guardian consent was provided for participants of school age.

At the interview, the authors obtained consent from participants and assured them of their rights, stating they were not obligated to participate and could withdraw at any time without consequences. Participants were given pseudonyms for anonymity.

### Data analysis

Interviews were audio-recorded with the participants’ permission. Data were transcribed verbatim, deidentified, and analysed using inductive content analysis [25]. All interviews were open coded by JH. The first interview was independently open coded by JH and IS, and any disagreements resolved by JD. JH clustered the codes into categories, which were then discussed with IS, and any disagreements resolved through discussion with JD to ensure validity and reliability [26]. The categories were clustered into sub-themes, and sub-themes into themes, capturing data relevant to the research question and representing the meaning and response within the dataset [27].

IS and JD undertook researcher triangulation, overseeing each stage of the analysis to reduce researcher bias, increase reliability and validity, and ensure credibility [28]. Experts in genetics and intellectual disability contributed to the validation of the categories, sub-themes and themes, including the three co-researchers (JLR, SS) [28].

## Results

### Participants

Three Australian high school students and 11 recently graduated young adults with intellectual disability participated (Table 1): seven males, five females and two non-binary participants, aged 12–28 years (average 24 years). Most participants attended public or Catholic schools with a mixture of mainstream or support classes. Interviews lasted from 8–73 min (average 34 min).

**Table 1:** Participant demographic data.

Name (pseudonym)	Age	Gender	Location (state) and type of school
Sonny	25	Non-binary	Public co-educational support unit (NSW)
Lee	28	Non-binary	Public co-educational mainstream and support classes (NSW)
Andrew	24	Male	Public co-educational support unit (NSW)
Jasmine	20	Female	Public co-educational support unit (NSW)
Michael	25	Male	Catholic mainstream single sex and support classes; public co-educational mainstream and support classes (NSW)
Duncan	15	Male	Catholic mainstream single sex and support classes (NSW)
Rodney	25	Male	Catholic mainstream single sex and support classes (NSW)
Lucas	26	Male	Catholic mainstream single sex and support classes (NSW)
Gail	20	Female	Public co-educational mainstream (NSW)
Jazzy	25	Female	Independent school for specific purposes (Victoria)
Bobby	24	Male	Public co-educational school for Specific Purposes (NSW)
Imogen	28	Female	Public mainstream co-educational and support classes (NSW)
Abigail	13	Female	Public mainstream co-educational and support classes (NSW)
Milo	12	Male	Public mainstream co-educational (NSW)

*NSW* New South Wales.

**Fig. 1:**
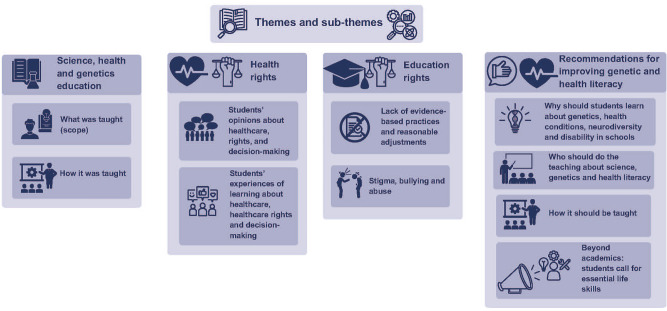
Overview of themes and subthemes.

Inductive content analysis revealed four key themes (Fig. 1): (i) Science, health and genetics education, (ii) Health rights, (iii) Education rights, (iv) Recommendations for improving education to support genetic and health literacy.

#### Science, health and genetics education

Participants commented on what was taught, how these topics were taught, and why they thought learning about these and related topics at school was important.

##### What was taught (scope)

Participating students had varying accounts of what they learned in science. They mentioned a wide range of topics, such as the difference between living and non-living things, planets, and chemicals. In health, they learnt about human anatomy, what humans need to survive, handwashing, and dental hygiene.

Ten participants shared what they were taught about genes and genetic conditions, which was highly variable. Sonny explained they had learned about genes but not genetic contributions to eye and hair colour.

Jazzy shared she had learned about genes and genetic conditions and connected it to something “health-wise”. She recalls being taught “there’s lots of genes in people” but that was not related to her health or that of others. However, she wondered about the connection between her genes and her own health and learning abilities “… I know there’s a disability in me, so obviously there’s something… a change”.

Four participants stated they did not learn about genes or genetic conditions at school. Andrew said, “I’d never heard of it”, and only when he started working at a self-advocacy organisation “that’s when I started to find out”. Andrew’s experience was similar to Sonny’s, who learnt about connections between genes and disability when they accessed School Leaver Employment Supports and started to question their parents: “It was up to me to be curious”.

##### How it was taught

Participating students identified several enablers to learning about science, health and genetics. These included experiments, outdoor learning experiences, videos, and checklists. Lucas summarised how teachers could facilitate learning to ensure students experience success: “Teacher taught, then video taught, then sign taught [signs and symbols], and movie taught”, because this sequence of steps would encourage students to attempt tasks independently. Several barriers to learning were also identified. Six participants had experienced inaccessible lessons, including a lot of information to recall and process, or where scientific vocabulary was not explained so that two participants felt “stupid”. One participant found equipment difficult to use, and their understanding and engagement were hindered by a lack of explanation.

For genetic concepts, students identified some enablers. Lucas described how teachers used videos, diagrams, signage, charts and worksheets to explain about genes and genetic conditions. One video showed if your father has a heart attack or diabetes “… you have a chance of having that as well”. Barriers to learning about genetic concepts included lessons that used animal examples instead of humans, or where students were not taught about genetic concepts at all.

#### Health rights

Participants shared their opinions about and experiences of healthcare, rights, and decision-making.

##### Students’ opinions about healthcare, rights, and decision-making

Students shared their opinions about the importance of learning about healthcare rights and making informed decisions. Eight participants described learning about health and healthcare choices as fundamental. Andrew thought that school “failed” to teach students about healthcare choices or to provide information on how to be informed health consumers. He noted the importance of learning about health and healthcare choices at an early age to prepare for adulthood, and highlighted the need for more teacher training, and resources on how to teach students about health and healthcare choices. Sonny felt they had “no control” over their body and “your body is there for other people to look at”. Participants had been taught that doctors have authority, and to accept whatever healthcare professionals say. Both Gail and her mother reported she was not taught to ask questions of doctors or make decisions about taking medication.

Andrew said schools should teach students about decision-making and choices as “… it’s important because you don’t want to go throughout adulthood always relying on the opinions of others”. He discussed how “we are our own individual selves” and “we have our right to be able to make decisions… according to our own needs”. Lee thought similarly, adding that not everyone has a support person throughout their whole life.

Two participants wished decision-making had been taught at school. Andrew recommended students be taught how to say “no” when they are uncomfortable with the presented choices presented, while Sonny suggested schools could support students to gain more confidence and experience with decision-making, for example, by providing ‘scripts’.

##### Students’ experiences of learning about healthcare, healthcare rights and decision-making

Education on healthcare rights and decision-making was very limited. Sexual health and puberty were topics recalled by participants, but they reported varied and fragmented experiences. Only one participant, Andrew, reported being taught about consent, while Sonny recalled that lessons about romantic experiences focused on heterosexual relationships. Imogen learned about respectful behaviours in romantic relationships. Two participants described learning about sexual relationships and the use of contraception to prevent sexually transmitted diseases. Four participants had been taught about the menstrual cycle and sanitary options.

Learning how to live healthily by eating well was highlighted by eight participants, whilst five participants mentioned keeping active. Two participants shared experiences of learning about mental health. Andrew had only been taught about severe depression; similarly, Sonny was only told about “extreme” mental health conditions in “very clinical” terms.

Only one participant, Rodney, was taught how to take medications safely by always seeking medical advice. Sonny had learned about recreational drugs and how you should “never do drugs”.

#### Education rights

Participants communicated the barriers they experienced to access quality education, lifelong learning opportunities, and dignity to pursue learning in a safe environment.

##### Lack of evidence-based practices and reasonable adjustments

Many barriers to learning were identified by participants. Curiosity was discouraged, as indicated by Sonny, who was removed from a class for asking too many questions. Repetition of the same content over several years, copying teachers’ notes from the board, and inaccessible school exams also hindered students’ interest and engagement.

Participants reported varying levels of support and reasonable adjustments. The lack of accessible and explicit instructions made it difficult for participants to complete tasks. Imogen and Abigail noted how discouraging it was when tasks were too hard or included too much reading. Being punished when they did not understand tasks was another barrier. Lee commented that insufficient time and support were provided: “they were always in a rush, and they never seem to care”.

##### Stigma, bullying and abuse

Bullying was a “sad reality” in high school for half of the participants. Lucas said he wished school taught more effective ways of dealing with bullying. Bobby implemented the strategy taught at primary school in high school, which was saying “Stop it, I don’t like it” but found it was ineffective: “Me say stop, and me move away, but they keep following me”. Jazzy wished she was taught how to make complaints to teachers about bullying.

Two participants shared how their traumatic high school experiences impacted them. Bobby highlighted that one of his teachers showed no understanding of students with disability, being “always mean”, raising her voice and calling students names.

Sonny reflected on their experience of unhelpful reactions to disclosures of abuse. When linking their experience of abuse to the school curriculum, Sonny wished they had been taught about recognising and reporting abuse, and that teachers need more training and skills of how to teach recognising and reporting abuse.

#### Recommendations for improving education to support genetic and health literacy

Students discussed why information about genetic conditions, health, and related topics of disability and neurodiversity should be taught at schools and made recommendations about who should be involved in teaching, how they would like to have received health and science education, and other topics related to health literacy that should be covered.

##### Why students should learn about genetics, health conditions, neurodiversity and disability in schools

Four participants discussed why people should learn about genes and genetic conditions. Jazzy highlighted the importance of learning about these topics as people “… want to know more… about something that they’ve got inside of them”.

Sonny thought that learning about genes and genetic conditions would encourage people to be “… a lot more accepting…” whilst Andrew highlighted that “… it gives people more of that understanding about the different types of disabilities… [and so they] … gain more compassion…” He noted “…there’s still a lot of stigma out there”.

Jasmine wished her school taught about Down Syndrome, but preferred people to use the term Trisomy 21 rather than Down Syndrome “because that makes me upset”. Sonny noted they were never taught about attention deficit hyperactivity disorder or autism at school. They wished they had and added that if autism continues to be a “taboo topic” in schools, people are going to associate neurodiversity with being “something bad”.

##### Who should do the teaching about science, genetics and health literacy

Imogen suggested that experts, such as doctors, be part of science instruction. Others suggested nurses, genetic healthcare and education professionals should be involved, as well as those with lived experience who could provide co-education.

Three participants described that teachers who understand students’ needs are significant enablers. Seven participants had access to teachers’ aides during lessons, with some positive experiences. Andrew said the teacher’s aide in his support class would break down tasks further than the subject teacher, and Sonny noted that support staff often had more knowledge of students’ reasonable adjustments than the subject teacher: “So the thing that stops learning is people who are not listening to the people [learning support staff] who know these kids the best”.

##### How it should be taught

Five participants gave examples of accessible ways to teach about genes and genetic conditions. All five suggested evidence-based practices, including visual supports and videos. Imogen suggested the GeneEQUAL Easy Read booklet about genes and genetic conditions as a resource for teachers [29].

##### Beyond academics: students call for essential life skills

Participants discussed the role of school in teaching them about decision-making and making choices more generally. Some participating students and young people were taught about decision-making in their daily lives, while others were not.

Three young people wished they had been taught more about emotions and emotional regulation. Sonny wished they were taught how emotions work, and Andrew wanted to have learnt more about compassion and understanding.

Sonny felt that schools need to teach about online information and social media more critically: “And schools have not kept up with any of that. That’s another reason why it doesn’t feel safe anymore”.

## Discussion

This study aimed to answer the research question: What are students’ perceptions and experiences of high school education relevant to health and genetics? The findings show participants received general education about sexual health and relationships, nutrition and physical activity, alcohol and illicit drug use, mental health and wellbeing, and occasionally about genes and genetic conditions. However, in general they did not receive information that linked clearly enough to their lives and supported them to make informed health decisions and be a partner in their own healthcare.

Health literacy skills involve reading, writing, speaking, listening, using technology, networking, and skills associated with making requests, advocacy, and complaints [30]. The barriers that most participants experienced to developing and consolidating health literacy skills included too much reading and complex information, lack of accessible and explicit instruction, difficult instructions, and complex scientific vocabulary. Under the Disability Standards for Education 2005 (Australian Government), teachers have a legal requirement to implement measures and actions to help students with disability access learning on the same basis as their peers without disability [31]. If students with intellectual disability are not taught health literacy skills at school, they risk lacking the knowledge needed to understand genetic testing results, evaluate treatment options, and participate actively in their healthcare decisions [14].

Poor health literacy is a significant public issue, as it hinders people’s ability to effectively communicate concerns, understand and follow health providers’ instructions, and benefit from health services [32]. This aligns with Australia’s National Roadmap for Improving the Health of People with Intellectual Disability, which identifies health literacy as a key priority area and calls for resources to ‘promote health literacy and advocacy skills among people with intellectual disability, their families and carers’ to support informed decision-making about health care [4]. Such poor health literacy has been directly linked to poor healthcare outcomes, and neglect, abuse and trauma in the healthcare system [2, 4]. Given that information about health and health systems is taught in schools [33], there is an urgent need to review how best to teach relevant curriculum content and upskill teachers in respectful, supportive, and trauma-informed teaching.

Participants recommended that explicit education about decision-making in general, and specifically about their own healthcare, should have been included in their school education [34]. If teachers are unaware of students’ needs and lack the professional development and resources to teach effective health literacy at schools, students are forced to rely on healthcare professionals, parents, carers, friends, and the media. However, our research and that of others clearly shows this does not happen consistently, due to multiple barriers including lack of time, resources and confidence of families and health professionals, as well as the assumption that people with intellectual disability lack the capacity to be partners in their healthcare [11, 15, 22, 35]. Without knowledge and skills, people are less likely to participate in making decisions about their healthcare and more likely to disengage from health screening and recommended therapies and lifestyle advice, which can be explicitly linked to higher rates of avoidable morbidities and death [36, 37].

The reality behind the school experience was a particularly disturbing theme. People with intellectual disability experience more abuse and violence than people without disability, with girls and women with intellectual disability experiencing higher rates of violence [2]. Being bullied, abused, and threatened are shared experiences of students with intellectual disability in education settings [38]. Globally, students with intellectual disability appear to be at higher risk of sexual abuse by peers or teacher/support staff [39]. Teachers may need additional support or resources to help them take into consideration students’ experiences of abuse and violence and how these affect learning and engagement with genetic health and health-related education. A trauma-informed framework has been identified as a critical component in effective education for students with intellectual disability, as evidenced by its inclusion in a recent State Parliamentary enquiry [40].

The participants also highlighted specific qualities they wished were taught and modelled at school: patience, understanding and compassion. These qualities were also communicated in a study exploring the ‘dream school’ for people with intellectual disability [38] and highlighted by students with and without disability in a study about the culture of their school, inclusion, and the practices implemented to support all students [41].

This is the first study asking students with intellectual disability about their perceptions and experiences of high school health and genetics education. By enrolling participants across different school sectors and support settings, our study provides a breadth of high school experiences.

A limitation of this research is the small number of participants and limited geographic spread (all but one participant attended a school in the state of New South Wales in Australia). As a result, perceptions and experiences are not necessarily generalisable to all students, and future studies should explore how a broader representative sample could be included equitably, including students with higher support needs and those from regional, rural and remote areas of Australia.

### Implications for future research

Future research conducted in other Australian states and territories and worldwide would capture broader perceptions and experiences of students and young people with intellectual disability. The principle of “Nothing about us without us”, and the shift towards a more inclusive socio-ecological model of disability, emphasise the importance of involving and valuing the opinions of students and young people with intellectual disability in educational settings [42]. Through collaboration, respect and empowerment, students with intellectual disability can become informed health consumers in adulthood [43].

While this study focused on students’ educational experiences, some participants mentioned questioning family members about genetic information or discussing health topics at home. Future research could explore how enhanced genetic and health literacy among students with intellectual disability might influence family communication and shared decision-making, potentially involving family members and siblings as participants.

Further research is needed in Australia to explore appropriate evidence-based practices to teach students with intellectual disability about health and genetic concepts. Strategies evaluated by researchers and identified as effective in educational settings [44] can then assist students to successfully access complex concepts, which will help them better understand their role and responsibility as informed health consumers.

Based on their recommendations, we propose that training for educators and resources (for example, lesson plans and multimedia resources) should be co-designed with people with intellectual disability and teachers, as well as other key stakeholders such as parents, using evidence-based approaches including trauma-informed strategies, visual supports and scaffolding techniques. These resources should focus on building foundational genetic and health literacy and skills and capability in shared decision making, self-management and self-advocacy. Such a package, especially if complemented by resources and training for healthcare professionals, has the potential to support more successful transitions from paediatric to adult health services. Collaboration with experts such as genetic healthcare professionals is needed to ensure accurate information and the development of person-centred and trauma-informed resources.

Our findings may inform health literacy initiatives in diverse international contexts, particularly in countries with expanding inclusive education programs or national genetic literacy goals. This positions our work within broader global conversations about inclusive education and health literacy development.

## Conclusion

This study highlights the importance of inclusive research in promoting visibility of people with intellectual disability. It helps to deconstruct ableism by valuing the lived experiences, opinions and contributions of people with intellectual disability as not only participants but as co-researchers [24]. We demonstrate how the voices and agency of students with intellectual disability, previously rarely elicited or heard, can be powerfully incorporated into much-needed changes in healthcare policy and practice.

Students in this study wanted to learn more about health and genetics in ways that would enable them to become informed health consumers. High schools have much to learn from the perceptions and experiences of students with intellectual disability, whose recommendations for accessible, person-centred and respectful approaches to health and genetic education can help prepare young people with intellectual disability to navigate the healthcare system as empowered partners in their own healthcare.

This will help prepare young people with intellectual disability to navigate the healthcare system and be empowered partners in their own healthcare. The growth of such skills has been suggested as critical in inequities in healthcare access for people with intellectual disability, as well as improving healthcare experiences and outcomes.

## Supplementary information

Easy Read summary

## Data Availability

Deidentified data are available upon request. Additional data and materials, such as data collection forms, data extraction and analysis templates can be obtained by contacting the corresponding author.
