# Supplementary material for: “It was up to me to be curious”: perceptions and experiences of students with intellectual disability on genetics and health education
Source: Eur J Hum Genet. 2026 Feb 23;34(4):491–7. doi: 10.1038/s41431-026-02041-w (PMC13046871; doi:10.1038/s41431-026-02041-w)
Supplement: Supplementary file 1 — Easy Read summary [file 41431_2026_2041_MOESM1_ESM.pdf]

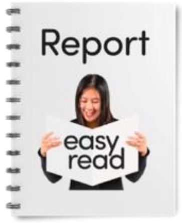

Health and genetic health taught in high schools

Our GeneEQUAL team wrote this report

In our GeneEQUAL team are

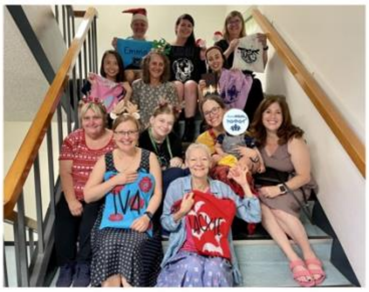

- people with intellectual disability
- researchers
- health care workers
- teachers of students with disability

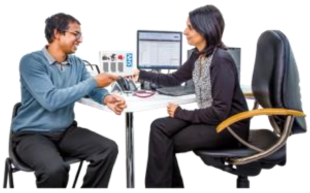

GeneEQUAL supports people with intellectual disability

- get better health care

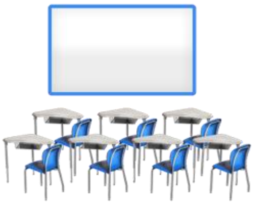

This report is about what students learn in school

- on their health and genetic health

What we know

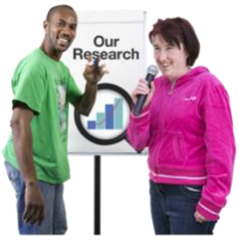

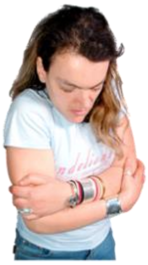

Many people with intellectual disability

- have bad experiences with health care
- do not have chances to make health care choices

People with intellectual disability said they want to learn

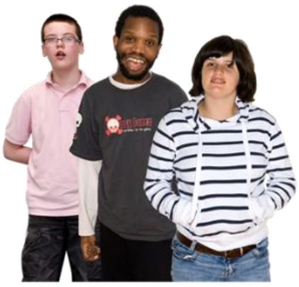

- about health and health care choices
- about health and genetic health

but school did not teach them

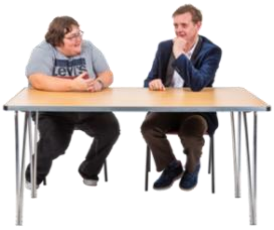

We need to find ways

- why these problems still happen
- how we can fix them

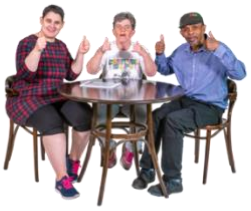

This way we can make health care better

- for people with intellectual disability

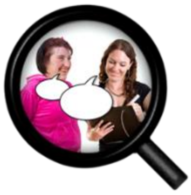

What we did

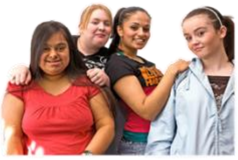

We talked to

- 14 students and young people with intellectual disability

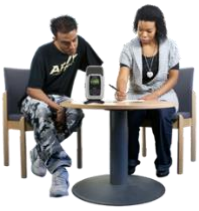

They told us about their experiences of

- health and genetic health taught at high school

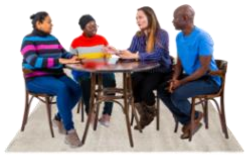

What we found out

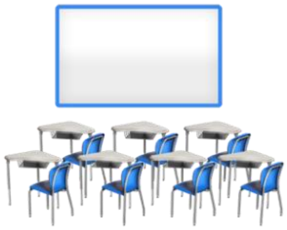

Students and young people with intellectual disability told us

- what was good and not good

- how to make health and genetic health education better

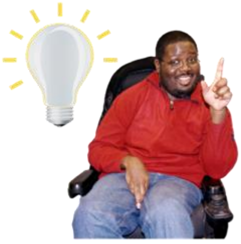

Idea 1
Learning about science, health, and genetics

Things that help students to learn science

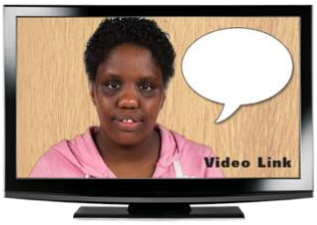

- experiments
- learning outdoors
- videos
- checklists

Things that make learning science hard

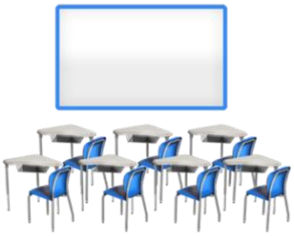

- too much information to remember
- teacher instructions that are hard to understand
- equipment hard to use

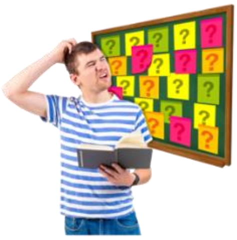

Hard words in science made people feel stupid

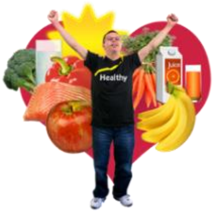

Most people learned about how to keep healthy

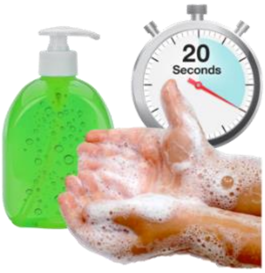

People learned

- how wash their hands
- brush their teeth

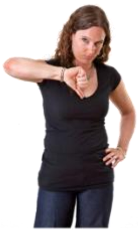

Not many people learned about genes and genetic conditions

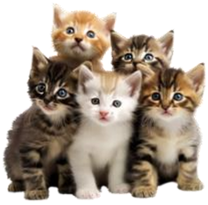

Genes were taught at school by talking about animals

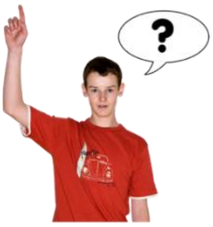

One person said

- it is up to me to be curious

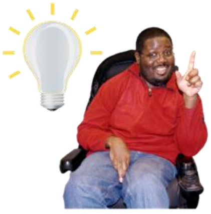

Idea 2
Health rights

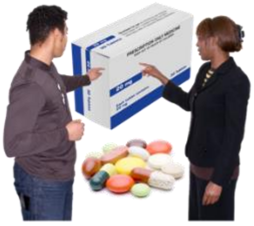

1 person learned how to take medication safely

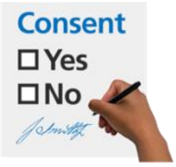

1 person learned about consent

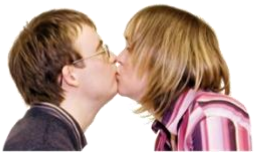

Students were taught at school only about

- romantic relationships between a man and a woman

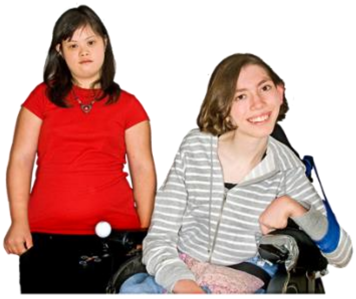

8 people said learning about

- health and health care choices is very important

It is an important skill for life

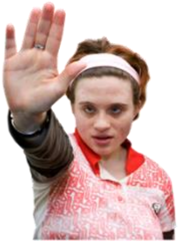

Schools could teach students how to make choices

- by teaching how to say ‘no’
- giving students scripts to follow

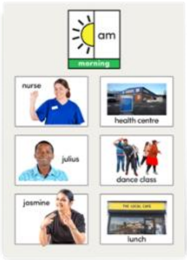

Schools should teach this by using

- Power Point and visual supports
- made-up stories of different situations

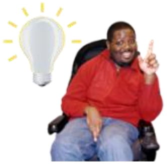

Idea 3
Education rights

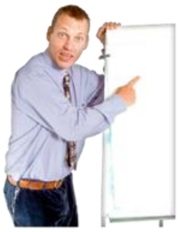

Things that make learning hard are

- copying notes from the board
- learning the same information each year

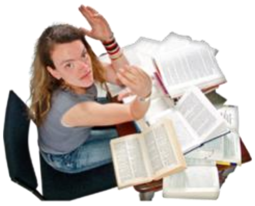

- doing hard tests
- too much reading
- difficult instructions

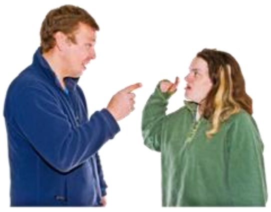

Teachers should not punish students

- for asking too many questions

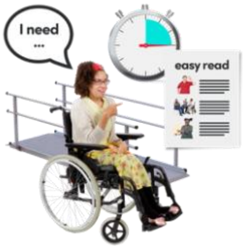

Teachers must use **reasonable adjustments**

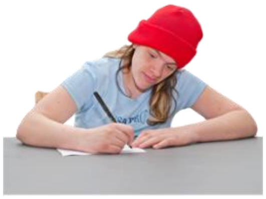

Reasonable adjustments are fair ways students can

- understand
- learn
- take part

in lessons

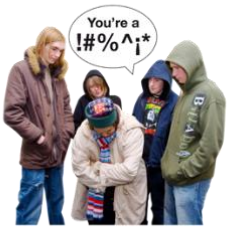

Nearly all people got bullied

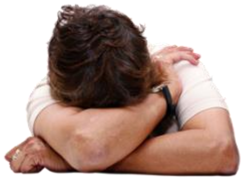

One person was abused

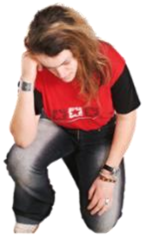

Most people did not feel good about themselves

- during high school

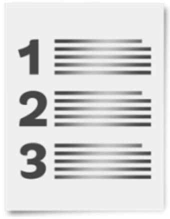

People had a long wish list

People wished high school taught better ways

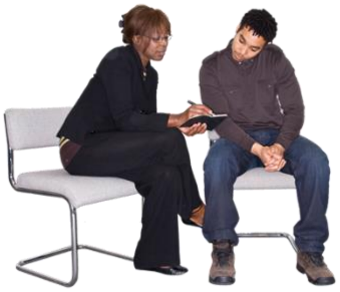

- to deal with bullying
- report bullying
- signs of abuse
- recognise and report abuse

Idea 4
How we can make things better

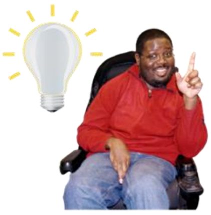

It is important to listen

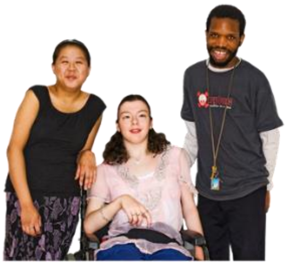

- to students and young people with intellectual disability
- what they think and go through at high schools

3 people said they wished

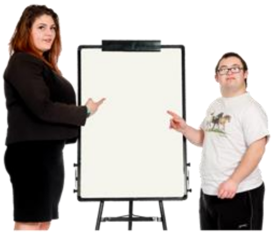

- schools taught about disabilities

1 person said schools need to teach about disability

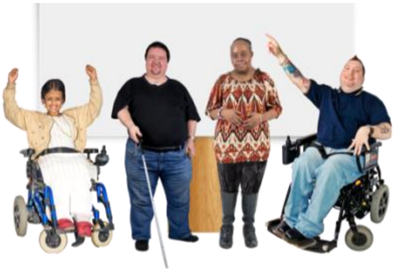

Otherwise people will think disability is something bad

People said they want to learn about genes and genetic conditions

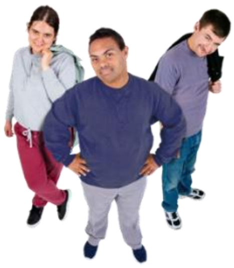

- by talking about people

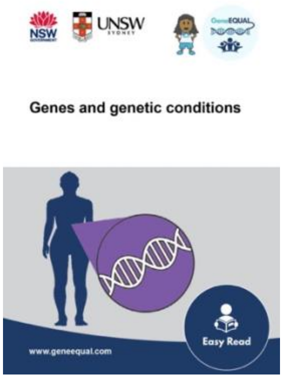

These things would help students to learn about genes

- visuals supports
- videos

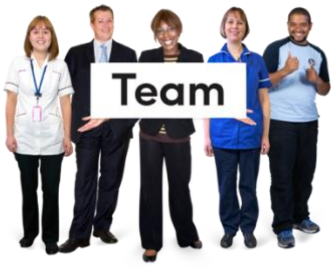

People said

- people with disability
- nurses
- doctors
- genetic health care workers

can help students learn better

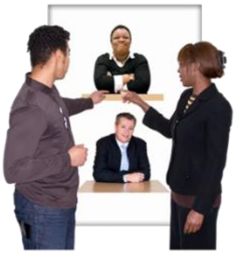

People said schools need

- to teach students how to make decisions

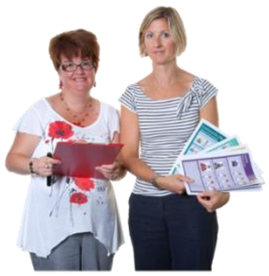

Schools must give teachers

- time
- training
- resources

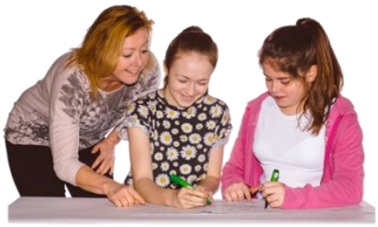

to help them teach students about

- health
- health care

making health care choices

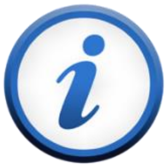

More information

This report was made with Photosymbols

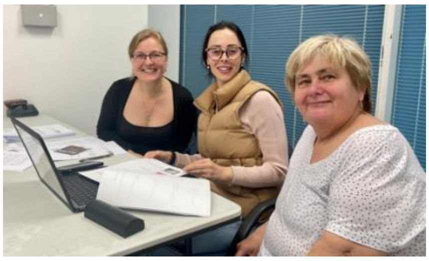

The full article is called

"It was up to me to be curious": Perceptions and experiences of students with intellectual disability on health and genetics education

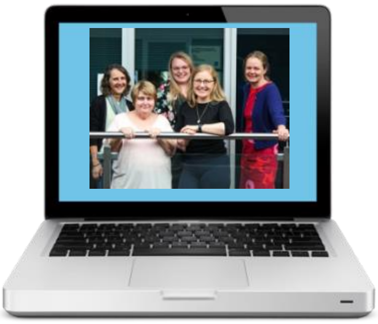

The GeneEQUAL website has

- the full article and more information

Go to

www.geneequal.com

or scan the QR code
